# A low-protein diet during pregnancy prevents modifications in intercellular communication proteins in rat islets

**DOI:** 10.1186/0717-6287-48-3

**Published:** 2015-01-16

**Authors:** Ana Flávia Marçal-Pessoa, Carmen Lucia Bassi-Branco, Cristiana dos Santos Barbosa Salvatierra, Luiz Fabrizio Stoppiglia, Letícia Martins Ignacio-Souza, Sílvia Regina de Lima Reis, Roberto Vilela Veloso, Marise Auxiliadora de Barros Reis, Everardo Magalhães Carneiro, Antonio Carlos Boschero, Vanessa Cristina Arantes, Márcia Queiroz Latorraca

**Affiliations:** Mestrado em Ciências da Saúde, Faculdade de Ciências Médicas, Universidade Federal de Mato Grosso, Cuiabá, Mato Grosso Brazil; Departamento de Ciências Básicas em Saúde, Faculdade de Ciências Médicas, Universidade Federal de Mato Grosso, Cuiabá, Mato Grosso Brazil; Departamento de Psicologia, Instituto de Educação, Universidade Federal de Mato Grosso, Cuiabá, Mato Grosso Brazil; Departamento de Alimentos e Nutrição, Faculdade de Nutrição, Universidade Federal de Mato Grosso, Cuiabá, Mato Grosso Brazil; Departamento de Anatomia, Biologia Celular e Fisiologia e Biofísica, Instituto de Biologia, Universidade Estadual de Campinas, Campinas, SP Brazil

**Keywords:** Connexin 36, Connexin 43, β-catenin, Low protein diet, Pregnancy, Pancreatic islets, Rat

## Abstract

**Background:**

Gap junctions between β-cells participate in the precise regulation of insulin secretion. Adherens junctions and their associated proteins are required for the formation, function and structural maintenance of gap junctions. Increases in the number of the gap junctions between β-cells and enhanced glucose-stimulated insulin secretion are observed during pregnancy. In contrast, protein restriction produces structural and functional alterations that result in poor insulin secretion in response to glucose. We investigated whether protein restriction during pregnancy affects the expression of mRNA and proteins involved in gap and adherens junctions in pancreatic islets. An isoenergetic low-protein diet (6% protein) was fed to non-pregnant or pregnant rats from day 1–15 of pregnancy, and rats fed an isocaloric normal-protein diet (17% protein) were used as controls.

**Results:**

The low-protein diet reduced the levels of connexin 36 and β-catenin protein in pancreatic islets. In rats fed the control diet, pregnancy increased the levels of phospho-[Ser^279/282^]-connexin 43, and it decreased the levels of connexin 36, β-catenin and beta-actin mRNA as well as the levels of connexin 36 and β-catenin protein in islets. The low-protein diet during pregnancy did not alter these mRNA and protein levels, but avoided the increase of levels of phospho-[Ser^279/282^]-connexin 43 in islets. Insulin secretion in response to 8.3 mmol/L glucose was higher in pregnant rats than in non-pregnant rats, independently of the nutritional status.

**Conclusion:**

Short-term protein restriction during pregnancy prevented the Cx43 phosphorylation, but this event did not interfer in the insulin secretion.

## Background

Pregnancy and a low-protein diet have opposing effects on insulin secretion. Pregnancy increases glucose-stimulated insulin secretion and reduces the threshold for stimulation of insulin secretion by glucose [1, 2]. This effect is attributed to enhanced glucose metabolism, increased activity of the cAMP and PLC pathways [1, 3–5], high β-cell proliferation and increased islet volume [6], insulin synthesis [7] and gap junction coupling among β-cells [8]. In contrast, a low-protein diet reduces insulin secretion in response to glucose as a result of structural and functional alterations, including the reduced size and/or volume of β-cells [9], a decreased level of coupling among β-cells possibly due to the low expression of connexin 36 [10], inappropriate glucose metabolism [11], diminished calcium handling [12] and alterations in the PLC (phospholipase C), PK (protein kinase) C and cAMP/PK (protein kinase) A pathways [13, 14]. The inability of pancreatic islets to increase insulin secretion to a sufficient level to compensate for insulin resistance during pregnancy due to protein restriction could contribute the development of gestational diabetes.

A large body of evidence has indicated that β-cell gap junctions are required for precise regulation of the biosynthesis, storage and release of insulin, particularly in response to glucose stimulation [15–18]. Connexin proteins form membrane channels at gap junctions, allowing β-cells to rapidly exchange cytoplasmic ions and metabolites, signaling the activity state of neighboring cells. This direct communication allows for a coordinated and synchronized response of the islet cell [16, 18–20]. The transcripts of at least three connexin isoforms (Cx36, Cx43, Cx45) have been repeatedly observed in extracts of intact pancreatic islets [21, 22] and purified β-cell preparations derived from these extracts [22, 23]. Immunolabeling studies have confirmed the expression of the Cx36 protein in the insulin-producing β-cells [16, 17, 22]. The pancreatic localization of the Cx43 protein has not been confirmed. However, an increase in Cx43 expression has been observed in rat neonatal islets exposed to prolactin, which promotes the secretory maturation of β-cells [21]. Evidence indicates that Cx43 can modulate cellular proliferation in a manner that is independent of gap junctional communication [24]. Both homozygous and heterozygous transgenic mice that overexpresses Cx43 present increases in the islet size, and heterozygous mice exhibit an increase in insulin levels [25].

Several studies have demonstrated the crucial role of the adherens junction and their associated proteins for the formation, function and structural maintenance of gap junctions [26, 27]. *In vitro* experiments have demonstrated a correlation between the expression of adhesion molecules, such as E-cadherin, and glucose-stimulated insulin secretion in the MIN6 β-cell lineage and sorted β-cell subpopulations [28–31]. β-catenin is an adherens junction-associated protein that links the cytoplasmic cadherin tail with the cytoskeleton (actin filament) and contributes to the function of cell adhesions [32–34]. A restriction in the level of maternal protein during pregnancy reduced β-catenin expression in placental vessels [35].

Decreased expression of Cx36 in β-cells is associated with increased [36–38] or reduced [39] basal insulin secretion, unaltered [36] or decreased [37] insulin secretion in response to high glucose concentrations, with impaired [39] or preserved [37] glucose homeostasis.

Interestingly, we previously verified [40] that islets from rats submitted to protein restriction during pregnancy exhibit an “inverted U-shape” dose–response curve, with elevated basal insulin secretion, a maximal insulin secretion in response to 8.3 mmol/L glucose, and blunted insulin secretion in response to 11.1 and 16.7 mmol/L glucose. It is reasonable to suppose that this secretory profile could, at least in part, to result from alterations in the expression of gap and adherens junction-associated proteins. Because the regulation of gap junction communication can occur at both the transcriptional and translational levels, we investigated the effect of protein restriction during pregnancy on the gene and protein expression of gap and adherens junction-associated proteins (Cx36, Cx43, β-catenin and β-actin) in pancreatic islets. This study is the first to describe the expression of these genes and proteins in islets from pregnant rats subjected to a low-protein diet. Evaluate the effect of protein restriction on molecular and cellular mechanisms involved in the β-cell adaptation during pregnancy could contribute to identify possibles cause of gestational diabetes and its prevention.

## Results

The low-protein non-pregnant (LPNP) group showed a higher food intake than the control non-pregnant (CN) group. Pregnancy enhanced the food intake in the two nutritional status groups, and the low-protein pregnant (LPP) group ate the same amount of food as the control pregnant (CP) group. Independently of nutritional status, pregnant rats had a greater body weight gain and a higher final body weight (F_1,66_ = 532.97, *P <* 0.0001 and F_1,66_ = 41.36, *P <* 0.0001, respectively), and they had a lower serum glucose concentration (F_1, 23_ = 5.80, *P <* 0.05) than the non-pregnant rats. The serum insulin concentration and the insulin:glucose ratio did not differ among the groups (Table 1).

**Table 1:** Nutritional, biochemical and hormonal profile in pregnant and nonpregnant rats that consumed control (CP and CNP) or low-protein diets (LPP and LPNP)

Variable	Groups			
	CN	CP	LPNP	LPP
Food intake (g)	203 ± 40^c^	282 ± 44^ab^	265 ± 18^b^	292 ± 37^a^
	(18)	(15)	(12)	(25)
Body weight gain (g)	27 ± 9	84 ± 12^#^	22 ± 5	80 ± 12^#^
	(18)	(15)	(12)	(25)
Final body weight (g)	266 ± 22	306 ± 24^#^	267 ± 15	307 ± 31^#^
	(18)	(15)	(12)	(25)
Serum glucose (mmol/L)	4.40 ± 1.50	3.10 ± 0.21^#^	3.93 ± 1.35	3.21 ± 0.67^#^
	(7)	(6)	(6)	(8)
Serum insulin (pmol/L)	137 ± 86	197 ± 132	160 ± 66	204 ± 156
	(7)	(6)	(6)	(8)
Insulin:glucose ratio	30 ± 14	65 ± 45	45 ± 23	63 ± 43
	(7)	(6)	(6)	(8)

Values are means ± SD for the number of rats shown in parentheses. Means with different superscript minuscule letters are significantly different by two-way ANOVA followed by a least significant difference (LSD) test (P < 0.05). ^#^Different in relation to non-pregnant rats (two-way ANOVA, *P* < 0.05).

**Figure 1:**
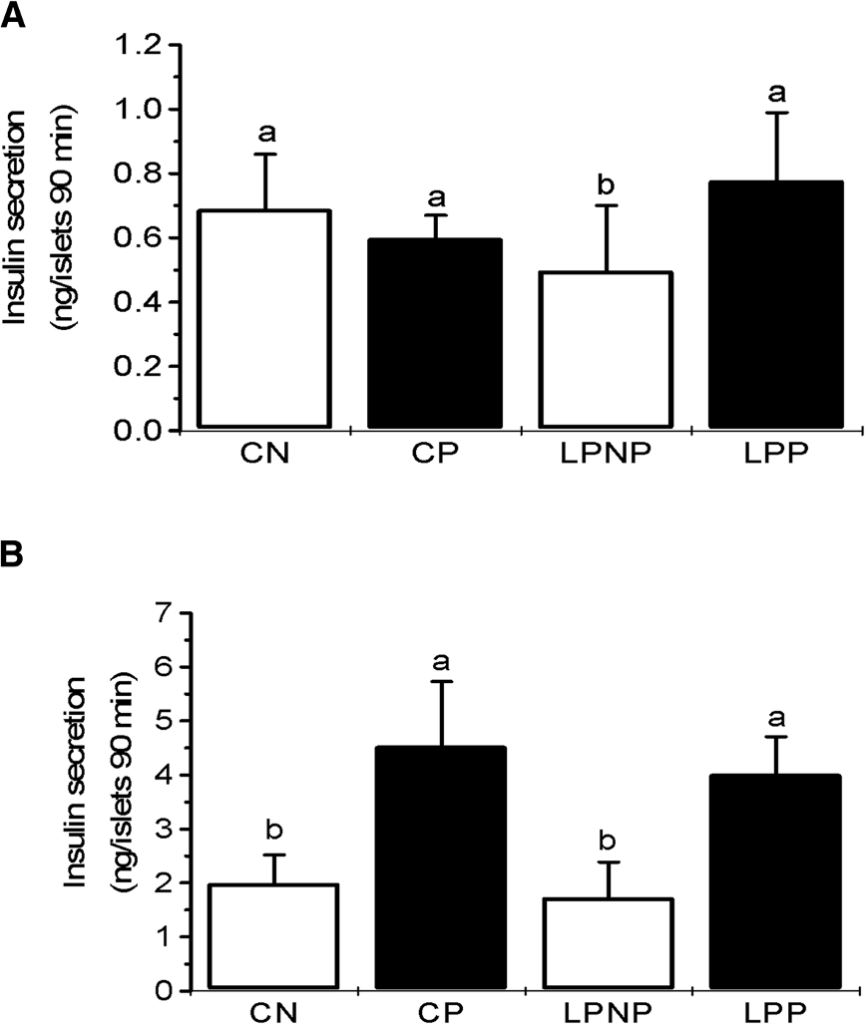
**Glucose stimulation of insulin secretion by islets from non-pregnant controls (CN), pregnant controls (CP), low-protein non-pregnant rats (LPNP) and low-protein pregnant rats (LPP).** Groups of 5 islets were incubated for 90 min in Krebs-bicarbonate medium containing **(A)** 5.6 or **(B)** 8.3 mmol/L glucose. The columns represent the cumulative 90-min insulin secretion and are the means ± SD of 5–9 independent experiments. Columns with different superscript minuscule letters are significantly different by two-way ANOVA followed by a least significant difference (LSD) test (P < 0.05).

In islets that were administered 5.6 mmol/L glucose, a two-way ANOVA revealed a significant effect of the interaction between the nutritional and physiological status (F_1,24_ = 8.03, *P* < 0.01). Thus, insulin secretion in the LPP, CP and CN groups was increased compared to that of the LPNP group (Figure 1A). Insulin secretion in the presence of 8.3 mmol/L glucose was influenced only by the physiological status (F_1,36_ = 90.13, *P* < 0.001); i.e., islets from pregnant (LPP and CP) rats released more insulin than islets from non-pregnant (LPNP and CN) rats (Figure 1B).

**Figure 2:**
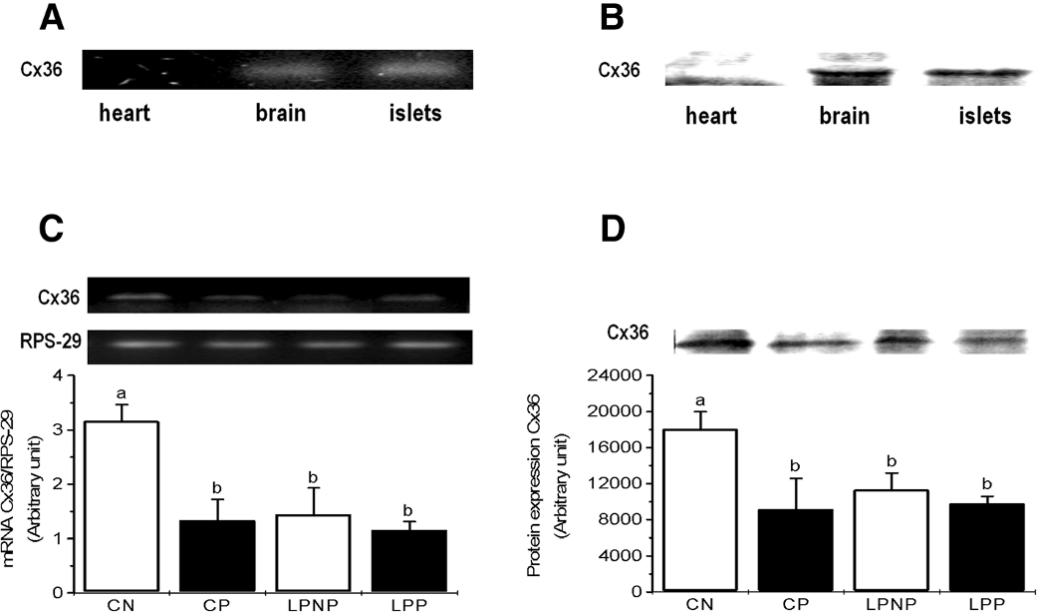
**Cx36 mRNA and protein expression in islets from non-pregnant controls (CN), pregnant controls (CP), low-protein non-pregnant rats (LPNP) and low-protein pregnant rats (LPP). (A)** and **(B)** Cx36 mRNA and protein expression in a heart sample (negative control), brain (positive control) and islets, respectively. **(C)** and **(D)** Cx36 mRNA and protein expression in islets from pregnant and non-pregnant rats fed control or low-protein diets. The Cx36 mRNA content was normalized to RPS29 mRNA. The columns represent the means ± SD of 3–5 independent experiments. Columns with different superscript minuscule letters are significantly different by two-way ANOVA followed by a least significant difference (LSD) test (P < 0.05).

Initially, the capacity for detecting Cx36 mRNA and protein expression was tested in tissues that are known to express high levels of this protein, such as the brain, and tissues known to express undetectable levels, such as the heart (Figure 2A and 2B).In LPNP islets, Cx36 mRNA and protein expression was lower than in CN islets. Pregnancy decreased the Cx36 mRNA and protein expression in CP islets, and Cx36 expression did not change in LPP islets. Thus, the expression of Cx36 mRNA and protein was similar in LPP, LPNP and CP islets (Figure 2C and 2D).

**Figure 3:**
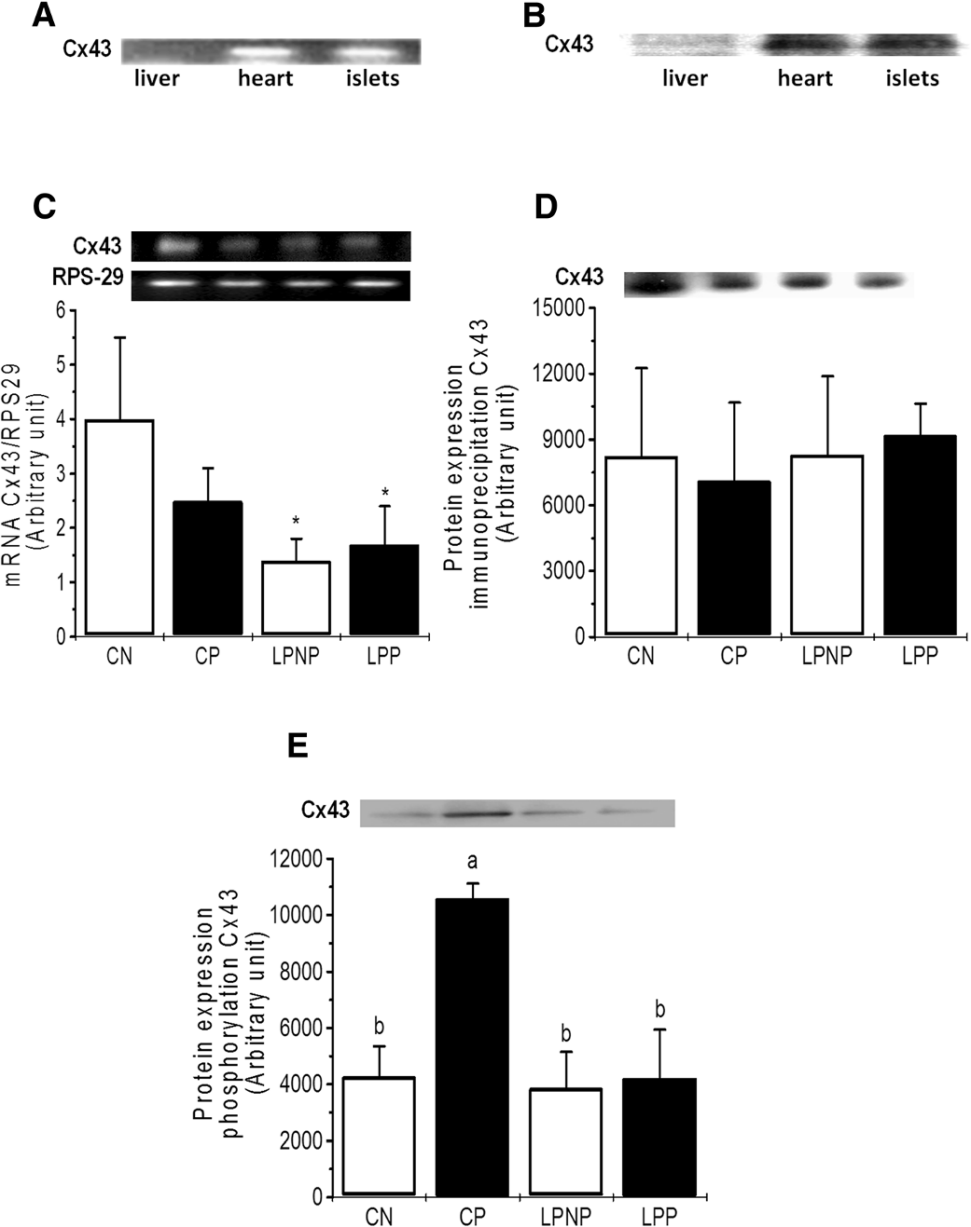
**Cx43 mRNA and protein expression in islets from non-pregnant controls (CN), pregnant controls (CP), low-protein non-pregnant rats (LPNP) and low-protein pregnant rats (LPP). (A)** and **(B)** Cx43 mRNA and protein expression in a liver sample (negative control), heart (positive control) and islets, respectively. **(C)** and **(D)** Cx43 mRNA and protein expression in islets from pregnant and non-pregnant rats fed control or low-protein diets. The mRNA concentration of Cx43 is expressed relative to RPS29 mRNA. (E) Phospho-[Ser279/282]-Cx43 content in islets from pregnant and non-pregnant rats fed control or low-protein diets. The columns represent the means ± SD of 3–5 independent experiments. Columns with different superscript minuscule letters are significantly different by two-way ANOVA followed by a least significant difference (LSD) test (P < 0.05).

The ability to detect Cx43 mRNA and protein expression was tested in liver (negative control) and heart (positive control), which express undetectable and high levels, respectively, of this connexin (Figure 3A and 3B). Cx43 mRNA expression was lower in LPP and LPNP islets than in CP and CN islets (F_1,10_ = 13.54, *P* < 0.01) (Figure 3C). The Cx43 protein content did not differ among the experimental groups (Figure 3D). The phospho-[Ser^279/282^]-Cx43 content was elevated in CP islets when compared to the other groups (Figure 3E).

**Figure 4:**
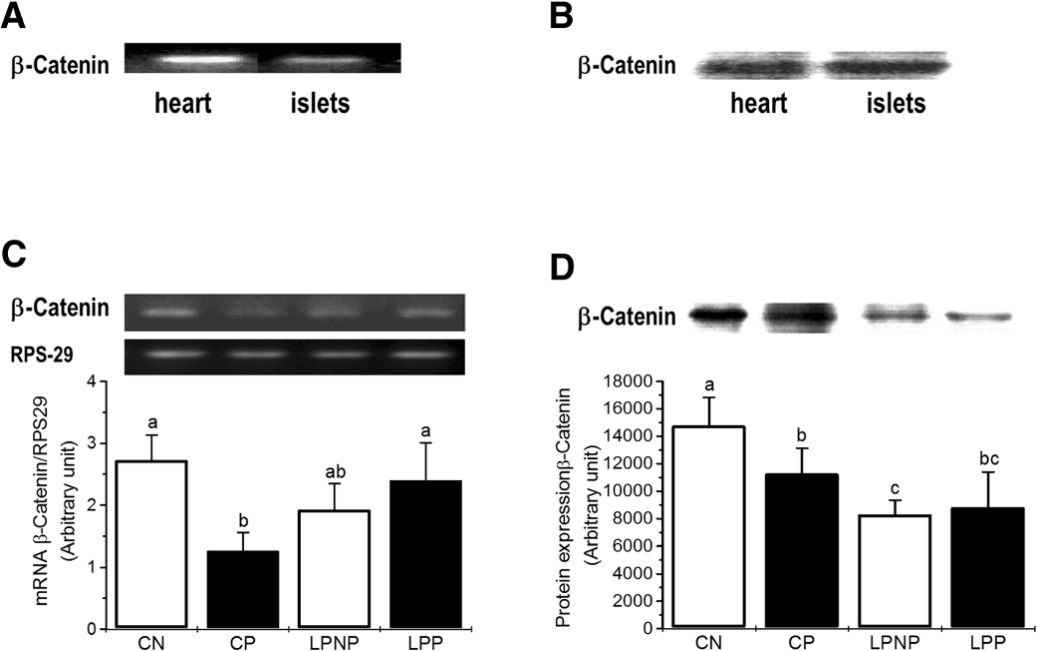
**β-catenin mRNA and protein expression in islets from non-pregnant controls (CN), pregnant controls (CP), low-protein non-pregnant rats (LPNP) and low-protein pregnant rats (LPP). (A)** and **(B)** β-catenin mRNA and protein expression in a heart sample (positive control) and islets. **(C)** and **(D)** β-catenin mRNA and protein expression in islets from pregnant and non-pregnant rats fed control or low-protein diets. The mRNA concentration of β-catenin is expressed relative to RPS29 mRNA. The columns represent the means ± SD of 3–6 independent experiments. Columns with different superscript minuscule letters are significantly different by two-way ANOVA followed by a least significant difference (LSD) test (P < 0.05).

As expected, we detected high β-catenin mRNA expression and protein content in a heart sample (Figure 4A and 4B). The expression of β-catenin mRNA (Figure 4C) and protein (Figure 4D) did not differ between LPP and LPNP islets and was reduced in the CP group compared to the CN group.

**Figure 5:**
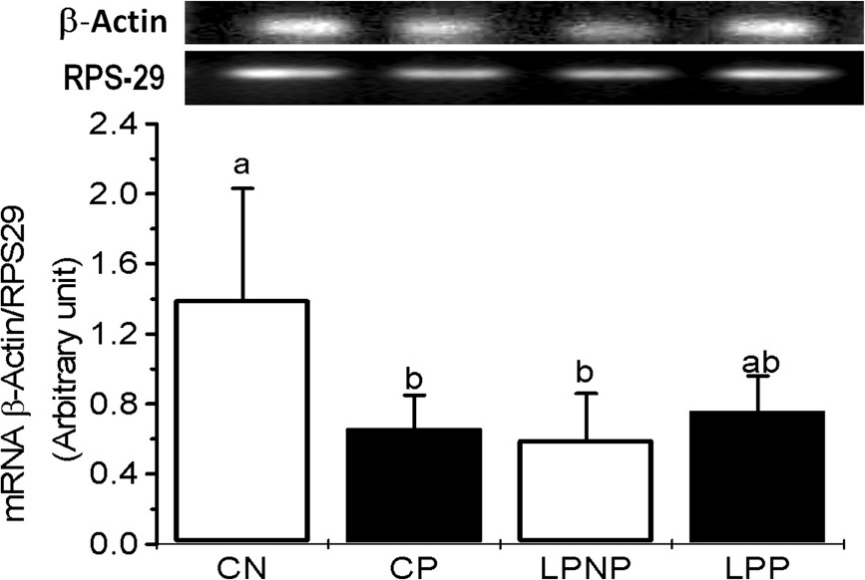
**β-actin mRNA expression in islets from non-pregnant controls (CN), pregnant controls (CP), low-protein non-pregnant rats (LPNP) and low-protein pregnant rats (LPP).** The mRNA concentration of β-actin is expressed relative to RPS29 mRNA. The columns represent the means ± SD of 3–4 independent experiments. Columns with different superscript minuscule letters are significantly different by two-way ANOVA followed by a least significant difference (LSD) test (P < 0.05).

Islets from the CP group exhibited lower expression of β-actin mRNA when compared with the CN group, and β-actin expression was similar in the LPP and LPNP islets (Figure 5).

## Discussion

In the present study, non-pregnant rats fed a low-protein diet exhibited increased food intake, in agreement with a previous report that mild protein restriction can result in hyperphagia [41]. Despite hyperphagia, the low-protein non-pregnant group ate half of the amount of protein ingested by control non-pregnant group. Pregnancy also produces hyperphagia [41–43], and the pregnant rats in this study exhibited a higher food intake that resulted in significant body weight gain and a consequent higher final body weight in comparison to the non-pregnant rats. Thus, a short duration of mild protein restriction during pregnancy did not alter the food-related behavior or somatic profile of the rats, corroborating previous observations [44].

Interestingly, protein restriction reduced the Cx36 transcript levels in pancreatic islets, and pregnancy reduced the Cx36 transcript levels in islets from rats maintained on the control diet. This pattern was confirmed by determination of the Cx36 protein content. Moreover, our data did not show a correlation between Cx36 expression and insulin secretion at physiological or basal glucose concentrations. Under physiological conditions, the pregnant rats exhibited higher insulin secretion than non-pregnant rats, which is in agreement with a previous study [2]. The unaltered insulin secretion exhibited by isolated islets incubated with 5.6 mmol/L glucose coincided with the unchanged basal serum insulin levels, although we observed a trend of increased insulinemia in both pregnant groups. Recently, we evaluated the kinetics of insulin release by isolated islets, and we verified that the mean level of insulin secretion during 20 minutes of perfusion with a low glucose concentration (2.8 mmol/L) was higher in pregnant rats, coinciding with basal hyperinsulinemia and low glucemia, regardless of the amount dietary protein (unpublished data). The hypoglycemia that is typical of normal pregnancy [45] was also observed in our pregnant rats. However, neither protein deprivation nor pregnancy altered the insulin:glucose ratio (which was used here as an indicator of insulin resistance), although the increase in the insulin:glucose ratio observed in pregnant rats approached statistical significance. Thus, Cx36 suppression in pancreatic islets from low protein non-pregnant rats and in islets from pregnant rats did not induce insulin resistance or glucose intolerance.

An important component of optimal long-term glucose homeostasis is the ability of the pancreatic beta-cell mass to vary its activity according to insulin requirements [46]. The beta-to-beta cell communication mediated by connexins is implicated in the regulation of the beta-cell mass during pregnancy [47], and Cx43 isoforms have been shown to increase the islet size or insulin content [25]. Thus, we investigated if protein restriction during pregnancy modulates Cx43 expression in pancreatic islets. We identified Cx43 in pancreatic islets, confirming the findings of other authors who showed that Cx43 is present in pancreatic islets cultured with prolactin [21] and that Cx43 is specifically expressed on the intra-islet endothelium rather than on β-cells [48]. Although we observed reduced levels of Cx43 mRNA in malnourished rats, the individual variations in the Cx43 protein levels were sufficient to mask the differences among our experimental groups. Additionally, the duration of exposure a low-protein diet may not have been sufficient to negatively modulate the translation process.

Cx43 is a MAP kinase substrate that, when phosphorylated on Ser^279^ and Ser^282^, disrupts gap junctions and initiates the down-regulation of gap junctional communication [49]. We found that the phospho-[Ser^279/282^]-Cx43 content was increased during normal pregnancy. However, the consumption of a low-protein diet during pregnancy did not result in an alteration of Cx43 phosphorylation, which was lower compared with the levels observed in islets from normal pregnant rats. The meaning of this result in pancreatic islets is still not clear; however, at least in epithelial cells, an increase in the phosphorylation of Cx43 appears to regulate its trafficking to the plasma membrane and its assembly into gap junctions [50]. Evidence exists that Cx43 phosphorylation may trigger its internalization and degradation [51]. The combined effects of phosphorylation and protein degradation by a proteasome-dependent mechanism contribute to the regulation of Cx43 stability in the plasma membrane and intracellular communication through gap junctions [52]. Lastly, in vascular smooth muscle cells, the proliferation is controlled through MAP kinase phosphorylation of Cx43 [53].

Important cellular processes, such as cell proliferation and growth, are partly regulated by connexin-cadherin interactions [54], and β-catenin plays a crucial role in cell adhesion in several epithelial cell types by modulating the linkage of cadherins to α-catenin, which in turn interacts with the actin cytoskeleton [55, 56]. Recently, it was shown that E-cadherin negatively regulates β-cell proliferation by reducing the levels of β-catenin in the nucleus, resulting in decreased D-cyclin levels [57]. In rodents, several lines of evidence suggest that prolactin (PRL) and/or placental lactogens (PLs) are responsible for the pregnancy-associated changes in the β-cell mass [58]. *In vitro* prolactin treatment induces higher β-catenin expression in islets cells, and high β-catenin correlates with increased Cx43 expression. We verified that in normal pregnancy, the β-catenin transcript levels and protein content were reduced, and no correlation was observed between the Cx43 protein and β-catenin content. However, our studies were performed on day 15 of pregnancy, and the levels of prolactin are known increase immediately before delivery [59]. We also observed that the decrease in the level of β-catenin mRNA correlated with low levels of β-actin mRNA during normal pregnancy. Thus, the profile of β-catenin protein expression as well as Cx43 phosphorylation observed in our normal pregnant groups coincides with the beginning of the decline of the proliferative phase of β-cells [58]. In contrast, the consumption of a low-protein diet during pregnancy did not alter the levels of β-catenin and β-actin transcripts; however, it did result in reduced β-catenin protein content, which had a level similar to the value observed during normal pregnancy. Considering the data on Cx43 phosphorylation and β-catenin together, it is reasonable to speculate that cellular processes that regulate the β-cell mass were reduced by the low-protein diet during pregnancy. However, these alterations did not contribute to an impairment of glucose tolerance.

## Conclusion

In conclusion, our results indicate that short-term protein restriction during pregnancy prevented the Cx43 phosphorylation, but this event did not interfere in the insulin secretion in the basal and physiological glucose ranges.

## Methods

### Animals and diet

The animal experiments were approved by the Institutional Committee for Ethics in Animal Experimentation (Universidade Federal de Mato Grosso). Non-pregnant Wistar rats (90 days old) were obtained from the university’s breeding colony. Mating was achieved by housing males with females overnight, and pregnancy was confirmed by the examination of vaginal smears for the presence of sperm. Pregnant and non-pregnant rats were each randomly assigned to two diet groups: control and low-protein. The control non-pregnant (CN) and pregnant (CP) groups were fed a 17% protein diet, and the low-protein non-pregnant (LPNP) and pregnant (LPP) groups were fed a 6% protein diet from days 1 to 15 of pregnancy. The diets were isocaloric, and the energy difference due to the reduction of dietary protein was compensated for by an equivalent change in the level of dietary carbohydrate, as described previously [40]. During the experimental period, the rats had free access to food and water and were housed at 22°C with a 12-h light:dark cycle. The food intake and body weight were recorded three times per week. At the end of this experimental period, the rats were weighed and killed by decapitation. Blood samples were collected and allowed to clot. Sera were stored at -20°C for the subsequent measurement of insulin by radioimmunoassay [60] and glucose by the oxidase-peroxidase system [61].

### Islet isolation and insulin secretion

The pancreas was removed from the rats and digested with collagenase type V (Sigma-Aldrich, CA, USA), as described elsewhere [62]. In the first series of experiments, groups of five islets were incubated for 90 min at 37°C in Krebs-bicarbonate buffer containing glucose (5.6 and 8.3 mmol/L) and equilibrated with a mixture of 95% O_2_ and 5% CO_2_ to result in a pH of 7.4. The incubation medium contained (in mmol/L): NaCl, 115; KCl, 5; CaCl_2_, 2.56; MgCl_2_, 1; NaHCO_3_, 24; and bovine serum albumin 3 g/L (Sigma-Aldrich, CA, USA). The insulin released was measured by RIA using rat insulin as a standard [60].

### Semiquantitative RT-PCR

Total RNA from 1,000 isolated and separated islets was extracted using TRIzol reagent (Life Technologies, Gaithersburg, MD). All of the reagents used in the experiments for *RT*-PCR were from Invitrogen (Carlsbad, CA, USA). For PCR analysis, RNA (2 μg) was reverse-transcribed using oligo (DT) primers. The resulting cDNA was amplified by PCR using oligonucleotides complementary to sequences in the Cx36 gene (5′- CGGTGTACGATGATGAGCAG -3′ and 5′- GAGTACCGGCGTTCTCTCTG -3′), Cx43 gene (5′-CCGACGACAACCAGAATGCC -3′ and 5′-CTTGGGATAGCTGGGCGGAAC-3′), β-catenin gene (5′-GCCAGTGGATTCCGTACTGT and 5′-GAGCTTGCTTTCCTGATTGC-3′), β-actin gene (5′-CAACCTTCTTGCAGCTCCTC-3′ and 5′-TTCTGACCCATACCCACCAT-3′) and RPS-29 gene (5′-CTGAAGGCAAGATGGGTCAC-3′ and 5′- CCATTCAGGTCGCTTAGTCC-3′). RPS-29 served as the internal control. The primers were from Prodimol (Belo Horizonte, MG, Brazil). The semiquantitative *RT*-PCR was performed in a 15-μL reaction volume containing 1 μL cDNA, 0.2 mM dNTP (dATP, dCTP, dGTP and dTTP), 1 mM MgCl_2_, 100% (v/v) 10 × PCR buffer, appropriate oligonucleotides primers (0.075 μM, 0.3 μM and 0.6 μM for RPS-29, Cx43, β-catenin, β-actin and Cx36, respectively) and 1 U *Taq* polymerase. The *RT*-PCR amplification conditions were as follows: for RPS-29 (internal control) and β-actin, 5 min at 94°C followed by 27 cycles of 45 s at 94°, 45 s at 59° and 1 min at 72°C; for Cx36, 5 min at 94°C followed by 35 cycles of 45 s at 94°, 45 s at 62° and 1 min at 72°C; for β-catenin, 5 min at 94°C followed by 27 cycles of 45 s at 94°, 45 s at 45° and 1 min at 72°C; and for Cx43, 5 min at 94°C followed by 30 cycles of 45 s at 50°, 45 s at 62° and 1 min at 72°C. The *RT*-PCR products were separated on a 1.5% agarose gel in 1 × Tris-borate-EDTA buffer and stained with ethidium bromide (USB Corporation, Cleveland, Ohio, USA). All of the assays included a negative control. The absence of contamination was confirmed by reverse transcriptase-negative RNA samples. The relative band intensities were determined by densitometry, and the ratio of Cx36, Cx43, β-catenin and β-actin to RPS-29 gene expression was calculated for each sample.

### Western blotting

After isolation, groups of islets were pelleted by centrifugation (15,000 x *g*) and then resuspended in 50–100 μL of homogenization buffer containing protease and phosphatase inhibitors [63, 64]. The islets were sonicated, and the total protein content was determined with a biuret (Labtest Diagnóstica, Lagoa Santa, MG, Brazil). Samples containing 200 μg of protein from each experimental group were incubated for 1 h at 37°C with 4 × concentrated Laemmli sample buffer (1 mmol sodium phosphate/L, pH 7.8; 0.1% bromophenol blue; 50% glycerol; 10% SDS; 2% mercaptoethanol) (4:1, v:v) and assayed on 12% polyacrylamide gels at 120 V for 90 min. The electrotransfer of proteins to nitrocellulose membranes (Bio-Rad) was performed for 2 h at 120 V in buffer lacking methanol and SDS. After checking the transfer efficiency by Ponceau S staining, the membranes were blocked with 5% skimmed milk in Tween-Tris-buffered saline (TTBS) (10 mmol Tris/L, 150 mmol NaCl/L, 0.5% Tween 20) overnight at 4°C. Cx36, phospho-^[Ser279/282]^-Cx43 and β-catenin were detected on the membranes after a 2-h incubation at room temperature with anti-Cx36 (goat polyclonal), anti-phospho-^[Ser279/282]^-Cx43 (goat polyclonal) and anti-β-catenin (mouse monoclonal) antibodies (Santa Cruz Biotechnology (Santa Cruz, CA, USA) and Zymed Laboratories (Invitrogen, CA, USA), respectively; diluted 1:500, 1:1000 and 1:1000, respectively, in TTBS containing 3% dry skimmed milk). To detect Cx43, samples containing 200 μg of total protein were incubated overnight at 4°C with 10 μL of anti-Cx43 mouse polyclonal IgG (Zymed, Invitrogen, CA, USA), followed by the addition of Protein A Sepharose (40 μL), transfer to nitrocellulose membranes, and blotting with a specific horseradish peroxidase-conjugated secondary antibody (Zymed, Invitrogen, CA, USA) diluted in blocking buffer (3% BSA 1:1500). Enhanced chemiluminescence (SuperSignal West Pico, Pierce) was used for detection. Band intensities were quantified by optical densitometry of the developed autoradiogram using Scion Image Beta software.

### Statistical analysis

The results were expressed as the mean ± SD for the number of rats (*n*) indicated. For islets, *n* refers to the number of experiments performed. Levene’s test for the homogeneity of variances was used initially to determine the fit of the data to the parametric ANOVA assumptions [65]. The data were analyzed by two-way ANOVA (nutritional status and physiological status). When necessary, these analyses were followed by LSD’s honestly significant difference test to determine the significance of individual differences. The level of significance was set at *P* < 0.05. The data were analyzed using the Statistic Software package (Statsoft).
